# Phenotype prediction from genome-wide association studies: application to smoking behaviors

**DOI:** 10.1186/1752-0509-6-S2-S11

**Published:** 2012-12-12

**Authors:** Dankyu Yoon, Young Jin Kim, Taesung Park

**Affiliations:** 1 Interdisciplinary Program in Bioinformatics, Seoul National University, Seoul, 151-742, Korea; 2 Center for Immunology and Pathology, National Institute of Health, Osong, Chungchungbuk-do, 363-951, Korea; 3 Center for Genome Science, National Institute of Health, Osong, Chungchungbuk-do, 363-951, Korea; 4 Department of Statistics, Seoul National University, Seoul, 151-742, Korea

## Abstract

**Background:**

A great success of the genome wide association study enabled us to give more attention on the personal genome and clinical application such as diagnosis and disease risk prediction. However, previous prediction studies using known disease associated loci have not been successful (Area Under Curve 0.55 ~ 0.68 for type 2 diabetes and coronary heart disease). There are several reasons for poor predictability such as small number of known disease-associated loci, simple analysis not considering complexity in phenotype, and a limited number of features used for prediction.

**Methods:**

In this research, we investigated the effect of feature selection and prediction algorithm on the performance of prediction method thoroughly. In particular, we considered the following feature selection and prediction methods: regression analysis, regularized regression analysis, linear discriminant analysis, non-linear support vector machine, and random forest. For these methods, we studied the effects of feature selection and the number of features on prediction. Our investigation was based on the analysis of 8,842 Korean individuals genotyped by Affymetrix SNP array 5.0, for predicting smoking behaviors.

**Results:**

To observe the effect of feature selection methods on prediction performance, selected features were used for prediction and area under the curve score was measured. For feature selection, the performances of support vector machine (SVM) and elastic-net (EN) showed better results than those of linear discriminant analysis (LDA), random forest (RF) and simple logistic regression (LR) methods. For prediction, SVM showed the best performance based on area under the curve score. With less than 100 SNPs, EN was the best prediction method while SVM was the best if over 400 SNPs were used for the prediction.

**Conclusions:**

Based on combination of feature selection and prediction methods, SVM showed the best performance in feature selection and prediction.

## Background

The main goal of genome-wide association studies (GWAS) is to identify the complex phenotype associated loci. A great success of the GWAS leads us to move our focus on the application to personal genomics and clinical practice such as diagnosis, disease risk prediction and prevention.

In personal genomics, one can genotype their own genome using direct-to-consumer (DTC) genotyping service provided by personal genomics companies such as 23andMe (https://www.23andme.com/) and DecodeMe (http://www.decodeme.com/). Personal genotyping will be followed by genome analysis and annotation for providing brief summary of genetic effects on various phenotypes of an individual. Moreover, SNPedia, a wiki based SNP database, can be used to expand the information regarding genetic effect of SNPs [1]. Personal genome data will be the baseline information for personal medical treatment and scientific research as the number of GWAS and genomics studies are growing. Personal genomics has already played an important role for scientific research. GWAS using personal genome data successfully have unveiled novel loci for common traits and Parkinson's disease [2,3].

In a clinical aspect, genetic analysis has provided valuable information for clinical treatment. For example, mutation analysis of *BRCA1 *and *BRCA2 *in breast cancer is valuable for clinical treatment [4,5]. Genotype information of *VKORC1 *and *CYP2C9 *can potentially be used as clinical information for estimating individual warfarin dose [6]. In genome-wide scale, Ashley et al. studied clinical usefulness of genome information [7].

The efforts described above are mainly focusing on interpretation of genomic information using previously identified phenotype associations. For diagnosis and clinical treatment, however, a more accurate phenotype prediction model is required. Previous studies have performed the disease prediction using phenotype-associated SNPs [8,9]. However, the prediction using previously known disease associated loci has not been quite successful [8,9]. For example, the prediction performance for type 2 diabetes and coronary heart disease using known associated loci have area under the curve (AUC) ranging from 0.55 ~ 0.68 [8,9]. There are three main reasons of this poor predictability [10]. Firstly, a limited number of previously known susceptibility variants were shown to explain only a small proportion of phenotypic variation [11]. Secondly, in previous studies, relatively simple statistical approaches were applied to GWAS data to explore disease associated loci. Additive mode of genetic inheritance and regression model were used, while not considering the complex relationships of interactions between multiple loci contributing to disease risk. Finally, genetic effect of variants would vary across phenotypes. Previous studies reported a wide range of heritability ranging from 0.2 to 0.99 [12]. A certain phenotype would be a result from the interaction of genetic and environmental effects.

There are two approaches to improve the poor predictability of phenotype based on the genetic variants. The first approach is to identify additional phenotype-associated loci and causal variants. A large scale genome-wide meta-analysis comprising tens of thousands individuals and next-generation sequencing technology are expected to unveil the hidden phenotype related loci. These methods would find more phenotype-associated loci and causal variants. Although this approach is promising, it requires a relatively high cost. The second is to develop a more accurate and reliable prediction method. In general, the prediction procedure consists of two steps: feature selection and prediction. Most previous efforts on disease prediction have largely focused on improving the performance of the prediction methods.

Moreover, most studies used a set of SNPs selected by p-values of simple linear regression model [8,9,13]. Low prediction performance of previous studies may partly be due to a failure to include genetic variants with complex relationship with phenotype. Recently, Wei et al. reported that prediction performance is varied by the number of variants used [10], demonstrating that selection of number of variants for the prediction is important to predict the risk of phenotypes. Single SNP analysis may not be adequate for identifying multiple causal variants and predicting risk of disease [14]. However, only a few studies discussed about the variable selection methods on GWAS data despite the importance of feature selection [13,15].

In this study, we investigated the effect of feature selection on the performance of the prediction methods more thoroughly. In particular, we considered the following methods for feature selection and prediction: logistic regression, linear discriminant analysis, regularized regression analysis, support vector machine, and random forest. For these models we studied the effect of feature selection on the performance of prediction and suggested an optimal number of features for improving the predictability of phenotypes. Our investigation was based on the analysis of GWA dataset of 8,842 KARE samples, for predicting smoking behaviors.

## Methods

### Data

Dataset was obtained from the Korea Association REsource (KARE) project as a part of Korea Genome Epidemiology Study (KoGES). Briefly, 10,004 samples were genotyped using Affymetrix Genome-Wide Human SNP Array 5.0. After quality control for samples and SNPs, 8,842 samples and 352,228 SNPs remained for subsequent analysis. The detailed information has been described in the previous studies [16]. In this analysis, we only used male samples for predicting smoking behaviors, because there are insufficient numbers of female smokers. Among 4,183 males, there were 807 individuals of non-smokers, 1,293 individuals of former and 2,164 individuals of current smokers. For number of cigarettes smoked per day (CPD) among smokers, KARE provides samples of 441 (CPD ≤ 10), 1,179 (11 ≤ CPD ≤ 20), 209 (21 ≤ CPD ≤ 30), and 129 (CPD ≥ 31). Given the smoking status, we defined three dichotomous traits such as smoking initiation ("never smoked defined as controls" vs. "former, occasional, or habitual smoker defined as cases"), CPD10 ("light smoking as controls with CPD ≤10" vs. "heavy smokers with CPD > 20 defined as cases"), and smoking cessation (SC) ("former smoker defined as controls" vs. "current smoker defined as cases"). Smoking behavior phenotypes are summarized in Table 1. The association results between the smoking behavior phenotypes and the SNPs have been reported in the previous studies [17-19].

**Table 1:** Smoking behaviours phenotypes

Phenotype	# of cases	# of controls
CPD10*	6,02	752
Smoking Initiation (SI)	3357	807
Smoking Cessation (SC)	2064	1293

CPD10: binary phenotype of nicotine dependence (ND) defined as <10 cigarettes/day and >21 cigarettes/day

For multiple SNP analysis, we often encounter information loss due to missing values. Therefore, we imputed missing genotypes using fastphase software [20] and obtained complete genotype data for multiple SNPs analysis. In particular, we imputed 8,842 samples with fastphase using options -T 10, -K 20 and -C 30.

### Feature Selection and risk prediction

Stronger statistical significance of SNP in the association analysis does not always assure a better disease risk prediction [21]. Moreover, most of previous prediction studies selected features based on the p-values from simple linear regression analysis. The emphasis on linear relationship between genotype and phenotype would omit the variants having complex relationship with phenotype. For studying the effect of feature selection on prediction performance, we used five statistical methods including logistic regression, linear discriminant analysis, penalized regression and data mining methods including support vector machine and random forest.

For feature selection, we used the scores computed by each prediction method to rank and select features. Considering complex relationship between variants and phenotype, simultaneous variable selection using the whole chromosome would be the most appropriate approach. Due to the immense amount of computation, however, we could not perform the analysis using the whole SNPs at the same time. Alternatively, the two step approach was used for the feature selection as suggested by Xu et al. [22]. First, all SNPs were partitioned into 22 chromosomal subsets. From each subset, feature selection was performed. Second, all SNPs were ordered based on their scores and 22,000 SNPs were selected for the additional joint feature selection. In this way, the SNPs showing the strongest association with the trait were selected for the subsequent prediction analysis. This two step approach was performed for each prediction method. We selected 22,000 SNPs due to limitation of computing resource in our laboratory.

For phenotype prediction, we used the same five prediction methods which were used for the feature selection step to observe the effect of combination of different methods in feature selection and prediction.

### Logistic regression

A logistic regression model is one of most widely used methods in the analysis of genomic data. Let ${\text{y}}_{\text{i}}\left(\text{i}=1,...,\text{n}\right)$be as a binary variable standing for the disease status (0 = control, 1 = case), and ${\text{x}}_{\text{ij}}\left(\text{j}=1,...,\text{p}\right)$defines as additive SNP value (0, 1, 2) according to the number of minor allele) for the jth SNP.

For feature selection, single SNP logistic regression (LR) analysis was conducted.

$$\text{log}\frac{\text{Pr}\left({\text{y}}_{\text{i}}=1\right)}{1-\text{Pr}\left({\text{y}}_{\text{i}}=1\right)}=\beta_{0}+\beta_{1}{\text{x}}_{\text{ij}}$$

where Pr(y_i _= 1) is the probability of subjects being cases (y = 1).β_0 _and β_1 _are the coefficients of intercept and SNP, respectively.

Multiple logistic regression (MLR) was used for prediction.

$$\text{log}\frac{\text{Pr}\left({\text{y}}_{\text{i}}=1\right)}{1-\text{Pr}\left({\text{y}}_{\text{i}}=1\right)}=\beta_{0}+\sum_{\text{j}=1}^{\text{k}}\beta_{\text{j}}{\text{x}}_{\text{ij}}$$

where k is the number of the selected SNPs in the feature selection step. β_0 _and β_j_'s are the intercept and effect sizes of SNPs, respectively.

### Elastic-Net Analysis

One caveat of using LR model in GWAS is that linkage disequilibrium (LD) dependency of input markers may make the parameter estimation unstable [10]. To address this issue, we imposed elastic-net regularization on the LR model building [23]. Elastic-net regularization uses ridge and LASSO penalties simultaneously to take advantages of both regularization methods. Thus, it provides shrinkage and automatic variable selection and can handle more efficiently with the severe multicollinearity that often exists in GWA analysis. Elastic-net regularization would perform better than LASSO in GWA analysis, in which multicollinearity persistently exists due to linkage disequilibrium among nearby SNPs [24]. Elastic-net regularization is particularly useful when the number of highly correlated predictor variables is much larger than the sample size. Elastic-net regularization solves the following problem:

$$\underset{\beta }{\text{min}}\left[\overset{\text{n}}{\underset{\text{i}=1}{\sum }}{\left({\text{y}}_{\text{i}}-\beta_{0}-{x^{\prime }}_{i}\beta \right)}^{2}+\lambda {\text{P}}_{\alpha }\left(\beta \right)\right],$$

where $x_{i}={\left({\text{x}}_{\text{i1}}\text{,}...\text{,}{\text{x}}_{\text{ip}}\right)}^{\text{T}}$ and $\beta ={\left(\beta_{1},\ldots ,\beta_{\text{p}}\right)}^{\text{T}}$. Elastic-net penalty is defined as ${\text{P}}_{\alpha }\left(\beta \right)=\left(1-\alpha \right)\sum |\beta |+\alpha \sum \beta^{2}$ where α is a weight of a value between 0 to 1. Cross validation (e.g., 10-fold) is generally employed to find the best values of λ and α, which minimize mean-squared prediction error [24]. Based on the result of EN, we selected SNPs with non-zero coefficients

### Linear discriminant analysis

Linear discriminant analysis (LDA) is used to find linear combinations of features which characterize or discriminate two or more classes. LDA is simple and fast. It often produces models with accuracy comparable to more complex methods [25]. LDA is the classifier that separates the two or more classes by determining the projection matrix that maximizes the ratio of between-class covariance to within-class covariance [25].

Linear discriminant function is

$$\text{L}\left(x\right)=x^{\text{T}}\Sigma^{-1}\left(\mu_{0}-\mu_{1}\right)-\frac{1}{2}{\left(\mu_{0}-\mu_{1}\right)}^{\text{T}}\Sigma^{-1}\left(\mu_{0}-\mu_{1}\right)+\text{log}\frac{p_{0}}{p_{1}}$$

Where $\mu_{0}$ and $\mu_{1}$ are means of the controls and cases, ∑ is common covariance matrix.

Feature selection is based on ranking SNPs by correlation-adjusted t (CAT) scores [26]. The cat score measures the individual contribution of each single feature to separate two groups, after removing the effect of all other genes.

### Support Vector Machine

Support vector machine (SVM) is a data mining approach for classification, regression, and other learning tasks [27,28], which shows empirically good performance and successful applications in many fields such as bioinformatics, text and image recognition. No assumptions are required about the underlying model. SVM finds an optimal hyperplane separating cases and controls and this process is based on large margin separation and kernel functions [27,28].

For function

f(x)=<ω,Φ(x)>+b

where Φ  is a mapping function of × to a high dimensional space, SVM find ω  and **b **such that ${min}_{w,\xi }\frac{1}{2}\omega^{T}\omega +C\sum \xi_{i}$ all $\left\{\left(x_{i},y_{i}\right)\right\}$, under the constraint ${\text{y}}_{\text{i}}(\omega^{T}x_{i}+b)\geq 1-\xi_{i}$**and **$\xi_{i}\geq 0$ for all i.

We used the radial-basis function as a kernel function.

$$\text{K}\left(x_{i},{x^{\prime }}_{i}\right)=\text{exp}\left(-\frac{∥x_{i}-{x^{\prime }}_{i}{∥}^{2}}{2\sigma^{2}}\right)$$

For SNP selection, SVM-RFE (Recursive Feature Elimination) algorithm for the variable selection task algorithm [29,30] is used. We used R statistics package e1071. For model building, we adopted default options including the radial kernel.

### Random Forest

Random Forest (RF) [31,32] is a classification algorithm using sets of random decision trees which are generated by a bootstrap sampling for decision and voting. Random subset of the variables is selected as the candidate at each split. RF has been widely used in pattern recognition and bioinformatics such as identification of gene and gene-gene interaction [33]. For feature selection, RF importance scores were used to rank and select SNPs. RF importance score is a measure for the relative contribution of a feature to the model.

### Cross-validation

The best strategy for performance comparison would be to use a separate validation set for accessing the performance of prediction models. However, the limited access to genomic data and phenotype information is often an obstacle to acquire a separate validation set. The second best strategy would be k-fold cross-validation comprising training data to fit the model and test data to measure the prediction performance.

In general, the whole data are split into k equal-sized subsets. Typically, selecting k is depending on the user's choice, usually 5 or 10 are recommended. For building prediction model, k-1 sets among k subsets are used as training set and the rest is used as a test set for measuring performance. This procedure iterates k times. Specifically, for i = 1,2,...,k the overall prediction performance is calculated from the k estimates of prediction performance [28]. In this study, 10 fold cross-validation was used to estimate the predictive performance for each prediction method.

### Prediction performance measure

For measuring prediction performance, we used the AUC of the receiver operating characteristic (ROC) curve that is most widely used method for measuring prediction performance [34,35]. ROC shows the relationship between sensitivity (true positive rate) and 1-specificity (false positive) at all possible threshold values. The AUC score is used as the indicator of discrimination power for binary traits. The score is ranging from 0.5 to 1 and higher score represents better discriminatory power.

## Results

**Figure 1:**
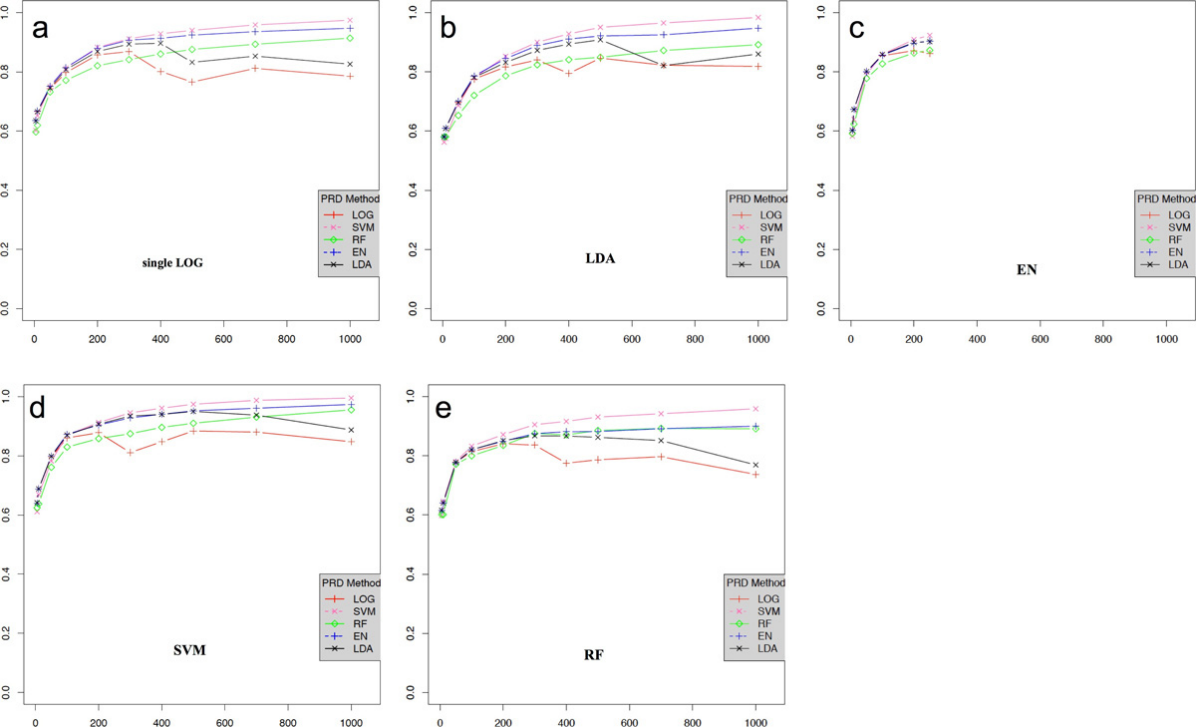
**Prediction performance according to feature selection method (a) logistic regression (LOG), (b) linear discriminant analysis (LDA), (c) Elastic Net (EN), (d) support vector machine (SVM), and (e) random forest (RF) varying # of SNPs used in prediction for CPD10**. X-axis represents the # of SNPs, Y-axis stands for the AUC score.

We compared the performance of the prediction methods such as logistic regression (LR), LDA, SVM, elastic net (EN) and random forest (RF). After the initial feature selection, we measured AUC of each prediction algorithm by increasing the number of features (5 ~ 1000 SNPs) used for the prediction. Performance comparison was based on 10-fold CV comprising 90% of samples for training set and rest 10% of samples for test set. AUC was calculated by averaging the performance scores of ten trials of cross validation. Figure 1 is the graph of prediction performance by various feature selection method varying the number of SNPs used in prediction for CPD10. Overall, the performance improves, as the number of features used for the prediction increases. LR and LDA, however, showed decreasing tendency in their performance when more than 300~700 variants were used for prediction. Although an increasing trend of performance was observed over the number of features, a quite large number of features would not be required to achieve the best performance. For example, the performance of each algorithm increases rapidly until the number of SNPs reaches 400 and then increase slowly, as shown in Figure 1.

Tables 2, 3 and 4 are the results of performance comparison with various feature selection methods for CPD10, SI and SC, respectively. Each column provides information about the performance of feature selection for a given prediction method, while each row does about the performance of prediction methods for a given feature selection method. In each column, the best performance among feature selection methods for a given prediction method is marked as underlined. In each row, the best performance of prediction methods for a given feature selection method is boldfaced.

**Table 2:** Performance results for CPD10

CPD10		Prediction method				
***Feature selection method***	***# of SNP***	***LR***	***SVM***	***RF***	***EN***	***LDA***

LR	100	0.7973	0.8128	0.7715	**0.8145**	0.8078
	400	0.8017	**0.9289**	0.8606	0.9137	0.8966
SVM	100	0.8605	0.8699	0.8295	**0.873**	0.87
	400	0.8474	**0.961**	0.8961	0.9405	0.9399
RF	100	0.8143	**0.8326**	0.7999	0.821	0.8206
	400	0.7752	**0.9164**	0.8709	0.8813	0.8669
EN	100^a^	0.8547	**0.8594**	0.8273	0.8567	0.8585
	250^a^	0.8621	**0.9235**	0.8731	0.9046	0.9022
LDA	100	0.7758	0.7801	0.7205	**0.7862**	0.7814
	400	0.7948	**0.9283**	0.8411	0.911	0.8939

In each column, the best results are shown as underlined. In each row, the best results are boldfaced.

**Table 3:** Performance results for SI

SI		Prediction method				
***Feature selection method***	***# of SNP***	***LR***	***SVM***	***RF***	***EN***	***LDA***

LR	100	0.7597	0.7171	0.7067	**0.7670**	0.7605
	500	0.8792	**0.9139**	0.8132	0.9038	0.8964
SVM	100	**0.6819**	0.6421	0.6204	0.6794	0.6813
	500	0.7953	**0.8178**	0.6930	0.7943	0.8075
RF	100	0.5961	**0.6101**	0.5980	0.5848	0.5957
	500	0.6185	**0.6312**	0.6138	0.6010	0.6210
EN	100^a^	0.7930	0.7708	0.7336	0.7929	**0.7937**
	163^a^	0.8157	0.8084	0.7454	**0.8188**	0.8180
LDA	100	0.6338	0.5925	0.5807	0.6273	**0.6343**
	500	0.7387	**0.7503**	0.6212	0.7176	0.7464

In each column, the best results are shown as underlined. In each row, the best results are boldfaced.

**Table 4:** Performance results for SC

SC		Prediction method				
***Feature selection method***	***# of SNP***	***LR***	***SVM***	***RF***	***EN***	***LDA***

LR	100	0.7290	0.7187	0.6919	**0.7372**	0.7308
	500	0.8245	**0.8644**	0.7856	0.8587	0.8591
SVM	100	**0.7417**	0.7299	0.6975	0.7413	0.7414
	500	0.8643	**0.8934**	0.8032	0.8780	0.8791
RF	100	0.7007	0.7053	0.7013	0.6992	**0.7008**
	500	0.7728	**0.8085**	0.7598	0.7676	0.7714
EN	100^a^	0.7682	0.7606	0.7233	**0.7691**	**0.7691**
	176^a^	0.7935	0.7964	0.7485	0.7967	0.7955
LDA	100	0.7015	0.6946	0.6585	0.7004	**0.7019**
	500	0.8205	**0.8466**	0.7426	0.8275	0.8291

In each column, the best results are shown as underlined. In each row, the best results are boldfaced. a. # of SNPs with non-zero coefficient.

For feature selection, the performance of SVM and EN showed better results than LDA, RF and simple LR methods. In overall, SVM showed the best performance for feature selection. SVM was the best with 400 and 500 SNPs for CPD10 and SC, while EN showed the best performance with 100 SNPs for SC (Table 2 and Table 4). As expected, simple LR and LDA did not perform well enough to explain complex relationship between SNPs and phenotypes. However, LR was not the worst in feature selection for SI and SC phenotypes. For SI phenotype, EN and LR were the best with 100 and 500 SNPs, respectively (Table 3).

These results imply that one feature selection method would not be always the best for various phenotypes. Thus, the prediction method seems to be carefully selected depending on the phenotypes.

For prediction, SVM outperformed other prediction algorithms for any feature selection method. Although SVM showed the best performance in overall, it was not the best method when a relatively small number of features were used. For example, LR, LDA and EN methods outperformed the SVM algorithm with features smaller than 400 for all phenotypes. With less than 100 SNPs, EN was the best prediction method while SVM was the best if over 400 SNPs were used for the prediction. For SI and SC phenotype, the results were similar to those of CPD10.

**Figure 2:**
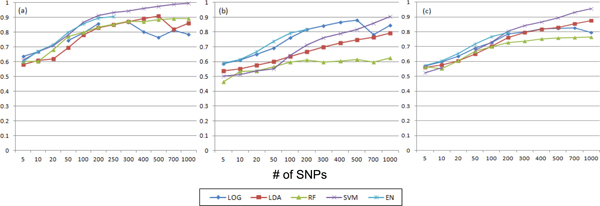
**Performance comparison with the same feature selection and prediction method (a) CPD10, (b) SI, and (c) SC**. X-axis represents the # of SNPs, Y-axis stands for the AUC score.

Figure 2 shows the performance results when the same method is used for feature selection and prediction. SVM was the best with more than 200 SNPs for CPD10 and SC (Figure 2 (a) and 2(c)). For SI phenotype, LR was the best with less than 700 SNPs while SVM outperformed LR with more than 700 SNPs (Figure 2 (c)). It is noteworthy that EN with less than 200 SNPs was the best for all phenotypes. We could not test EN with more than 300 SNPs because non-zero coefficient of SNPs from EN feature selection was less than 300 SNPs.

We investigated the best combination of feature selection and prediction algorithm. The best combination of feature selection and prediction algorithm with 100 SNPs were SVM-EN (AUC: 0.873 for CPD10), EN-LDA (AUC: 0.771 for SI) and EN-EN (AUC: 0.769 for SC). The best combinations for more than 400 SNPs, however, were SVM-SVM (AUC: 0.961 for CPD10), LR-SVM (AUC: 0.914 for SI), and SVM-SVM (AUC: 0.893 for SC).

In the current study, we examined the performance of prediction methods in combination of feature selection and prediction algorithm. All prediction methods showed the best performances when around 400~500 features were used. For prediction method, SVM with radial kernel outperformed the other methods regardless of algorithms used in the feature selection.

## Discussion

In this study, the performance of prediction methods was compared in combination of various feature selection and prediction algorithms. Also, the effect of the number of SNPs on prediction performance was investigated. In earlier study of Wei et al. [10] and Kooperberg et al. [15], limited studies of the prediction performance were presented. Wei et al. used SVM and logistic regression while Kooperberg et al. used logistic regression and penalized regression such as lasso and elastic net. Further, they have not discussed about the effect of various feature selection methods. Although Kooperberg et al. suggested penalized regression based feature selection during cross-validation using subset of data, they did not compare their results to other statistical methods such as SVM, LDA and RF which are used in our study. Our study thoroughly performed the comparison analysis of combinations of feature selection and prediction algorithms including logistic regression, EN, LDA, SVM and RF. Complex relationship between phenotype and genetic variants was considered by using various statistical methods. Therefore, our study is particularly valuable in the context of comprehensive comparison analysis for improving prediction performance by the various condition of the number of features, feature selection, and prediction algorithm. In addition, it is noteworthy that the feature selection in our study was conducted on genome-wide scale. This is an important difference in that the previous studies have a limitation in the feature selection, because it used the p-values from the simple linear regression method [8-10,13,15].

The performance of prediction method via AUC varied by phenotypes. The difference in magnitude of the prediction performance is dependent on the proportion of genetic effect on phenotypes [36]. For example, prediction performance for CPD10 was the best among smoking phenotypes. This is consistent with the level of heritability of smoking phenotypes. The estimated heritability was known to be about 0.59 and 0.37 for CPD10 and SI, respectively [37]. Based on these results, we may improve the prediction model by including the clinical variables for those of phenotypes with relatively low heritability.

Overall, SVM outperformed all other prediction methods in feature selection and prediction. Note that SVM is the most complex prediction algorithm. Thus, the good performance of SVM implies that complex relationship of genetic effect on phenotypes should be taken into consideration for selecting features and building a prediction model. However, it is also important to emphasize that SVM was not the best if a relatively small number of features were used for phenotype prediction. Therefore, feature selection and prediction algorithms should be carefully selected depending on the phenotypes. We first expected that prediction performance would be the best if the same algorithm is used for feature selection and prediction. However, our results indicated that a certain prediction methods did not provide the best fit for the features selected by the same algorithm. Set operations, like union, intersection, or majority voting, can be applied to the feature selection process. For example, a union set of all selected features from different methods can be used for prediction. Alternatively, the common features from two or more selection methods, defined as an intersection set, can be used for prediction. Majority voting approach which chooses the features selected by more than half of the methods can also be used. However, our application of set operations to KARE data did not improve the prediction results much (data not shown). A further systematic study on set operations is desirable.

For measuring the prediction performance, we adopted 10-fold cross-validation. Since we did within-study cross-validation, our performance measures may be overestimated. Independent genotype data may be required for complete assessment of prediction performance comparison. The ultimate goal of genome study is clinical practice based on personal genome. In this context, our prediction analysis using genomic information is important in order to understand human genome and apply it to clinical studies.

## Conclusions

In this study, we performed comprehensive comparison analysis for improving prediction performance by the various condition of the number of features and combination of feature selection and prediction algorithm including logistic regression, EN, LDA, SVM and RF. Overall, SVM outperformed other methods in feature selection and model prediction. With less than 100 SNPs, EN was the best prediction method while SVM was the best if over 400 SNPs were used for the prediction.

## Competing interests

The authors declare that they have no competing interests.

## Authors' contributions

DKY and TSP designed the study and DKY, YJK and TSP carried out statistical analysis. TSP coordinated the study. DKY, YJK and TSP wrote the manuscript. All authors read and approved the final manuscript.
